# Sex differences in risk factor relationships with subarachnoid haemorrhage and intracranial aneurysms: A Mendelian Randomisation study

**DOI:** 10.1177/23969873241265224

**Published:** 2024-07-30

**Authors:** Lena Tschiderer, Mark K Bakker, Dipender Gill, Stephen Burgess, Peter Willeit, Ynte M Ruigrok, Sanne AE Peters

**Affiliations:** 1 Institute of Health Economics; Medical University of Innsbruck, Innsbruck, Austria; 2 Department of Neurology and Neurosurgery, University Medical Center Utrecht Brain Center, Utrecht University, the Netherlands; 3 Department of Epidemiology and Biostatistics, School of Public Health, Imperial College London, London, United Kingdom; 4 Department of Public Health and Primary Care, University of Cambridge, Cambridge, United Kingdom; 5 Heart and Lung Research Institute, University of Cambridge, Cambridge, United Kingdom; 6 MRC Biostatistics Unit, School of Clinical Medicine, University of Cambridge, Cambridge, United Kingdom; 7 Julius Center for Health Sciences and Primary Care, University Medical Center Utrecht, Utrecht University, Utrecht, the Netherlands; 8 The George Institute for Global Health, School of Public Health, Imperial College London, London, United Kingdom; 9 The George Institute for Global Health, University of New South Wales, Sydney, New South Wales, Australia

**Keywords:** aneurysmal subarachnoid haemorrhage, intracranial aneurysm, sex differences, Mendelian randomisation

## Abstract

**Background:**

The prevalence of intracranial aneurysms (IAs) and incidence of aneurysmal subarachnoid haemorrhage (aSAH) is higher in women than in men. Although several cardiometabolic and lifestyle factors have been related to the risk of IAs or aSAH, it is unclear whether there are sex differences in causal relationships of these risk factors.

**Aims:**

The aim of this study was to determine sex differences in causal relationships between cardiometabolic and lifestyle factors and risk of aSAH and IA.

**Methods:**

We conducted a sex-specific two-sample Mendelian randomisation study using summary-level data from genome-wide association studies. We analysed low-density lipoprotein cholesterol, high-density lipoprotein cholesterol [HDL-C], triglycerides, non-HDL-C, total cholesterol, fasting glucose, systolic and diastolic blood pressure, smoking initiation, and alcohol use as exposures, and aSAH and IA (i.e., aSAH and unruptured IA combined) as outcomes.

**Results:**

We found statistically significant sex differences in the relationship between genetically proxied non-HDL-C and aSAH risk, with odds ratios (ORs) of 0.72 (95% confidence interval 0.58, 0.88) in women and 1.01 (0.77, 1.31) in men (P-value for sex difference 0.044). Moreover, genetic liability to smoking initiation was related to a statistically significantly higher risk of aSAH in men compared to women (P-value for sex difference 0.007) with ORs of 3.81 (1.93, 7.52) and 1.12 (0.63, 1.99), respectively, and to a statistically significantly higher IA risk in men compared to women (P-value for sex difference 0.036) with ORs of 3.58 (2.04, 6.27) and 1.61 (0.98, 2.64), respectively. In addition, higher genetically proxied systolic and diastolic blood pressure were related to a higher risk of aSAH and IA in both women and men.

**Conclusions:**

Higher genetically proxied non-HDL-C was related to a lower risk of aSAH in women compared to men. Moreover, genetic liability to smoking initiation was associated with a higher risk for aSAH and IA in men compared to women. These findings may help improve understanding of sex differences in the development of aSAH and IA.

## Introduction

Aneurysmal subarachnoid haemorrhage (aSAH) is a subtype of stroke that occurs after rupture of an intracranial aneurysm (IA).^1^ Sex differences in experiencing these subtypes are known^2^ as women are overrepresented in the aSAH population.^3^ For instance, in 2019, five million women had prevalent aSAH compared to three million men.^3^ Moreover, women have a higher risk for IA rupture than men.^4^

Several cardiometabolic and lifestyle factors have been related to the risk of aSAH. In traditional epidemiological observational studies, smoking and hypertension were associated with a higher aSAH risk, while hypercholesterolaemia and diabetes were related to a lower aSAH risk.^5^ Similarly, in a Mendelian randomisation study, smoking initiation and greater systolic blood pressure (SBP) and diastolic blood pressure (DBP) were causally linked to a higher risk for aSAH, whereas higher fasting glucose was related to a lower risk for aSAH.^6^ Another Mendelian randomisation study found higher low-density lipoprotein cholesterol (LDL-C) to be related to a lower risk of aSAH and unruptured IAs combined.^7^ Sex differences in risk factor associations with aSAH have previously been suggested by observational studies, which reported no differences in the association of hypertension, smoking, and hypercholesterolaemia with aSAH, but a significantly lower risk for aSAH associated with diabetes in women compared to men.^8^ However, sex-specific causal effects of these risk factors on aSAH have not yet been studied.

We performed a Mendelian randomisation study on the sex-specific relationship between genetically proxied cardiometabolic and lifestyle factors and the risk for aSAH and IA and quantified sex differences in these associations.

## Methods

The results of this work are reported according to the STROBE-MR Statement. The STROBE-MR checklist is provided in eTable 1.

### Genetic data

To estimate the causal relationship between cardiometabolic and lifestyle factors (exposures) and the risk for aSAH and IA (outcomes), we conducted a two-sample Mendelian randomisation study. In traditional observational studies, associations between exposures and outcomes can be affected by measured and unmeasured confounding. This means that findings can be driven by factors that are related to both the exposure and the outcome. Under certain assumptions, Mendelian randomisation overcomes this issue and allows estimating exposure-outcome relationships independent of confounding.^9^ Randomised controlled trials are the gold standard of estimating causal links between exposures and outcomes, randomising individuals into intervention and control groups. The concept of Mendelian relies on the random assignment of genes at the time of gamete formation and conception using single-nucleotide polymorphisms (SNPs) as instrumental variables, and in that regard, is comparable to a randomised controlled trial.^9^ To conduct a Mendelian randomisation study, genetic associations with both exposures and outcomes are required, which can, for instance, be based on results from genome wide association studies (GWASs).

For the present study, our instrumental variables included SNPs from four large-scale international GWASs associated with the following cardiometabolic and lifestyle factors: (1) lipid traits including LDL-C, high-density lipoprotein cholesterol (HDL-C), triglycerides, non-HDL-C, and total cholesterol from the Global Lipids Genetics Consortium (GLGC),^10^ (2) fasting glucose from the Meta-Analyses of Glucose and Insulin-related traits Consortium (MAGIC),^11^ (3) SBP and DBP from the International Consortium for Blood Pressure (ICBP),^12^ and (4) smoking initiation and alcohol use from the GWAS and Sequencing Consortium of Alcohol and Nicotine use (GSCAN).^13^ Genetic associations with lipid traits are reported per higher standard deviation, with fasting glucose per mmol/L increase, with SBP and DBP per 10 mmHg increase, with smoking initiation per unit increase in the log odds of ever versus never smoking, and with alcohol use per additional drink per week. To create sex-specific genetic instruments, we used genetic associations with exposures from sex-specific GWASs, conducted separately for women and men, if available. We used sex-specific genetic instruments for lipid traits and fasting glucose and sex-combined genetic instruments for SBP, DBP, smoking initiation, and alcohol consumption (as no sex-specific summary-level data were available). Whenever available, we used SNPs from individuals of European ancestry (for lipid traits, glucose, smoking initiation, and alcohol use).

As outcomes, we analysed aSAH (i.e., ruptured IAs) and IA (i.e., aSAH and unruptured IAs combined). Sex-specific genetic associations with aSAH and IA were obtained from a GWAS by Bakker et al., which included individuals of European ancestry.^14^ Although we performed two-sample Mendelian randomisation and used data from different GWASs for genetic associations with the exposure and outcome, they are not fully disjoint as there are studies, which were included in multiple GWASs. Increasing sample overlap in the data used to obtain genetic associations with the exposures and outcomes can introduce bias.^15^ To reduce sample overlap, we excluded UK Biobank data for genetic associations with outcomes when analysing lipid, smoking, and alcohol traits. This lowers over-fitting and any bias from weak instruments will be towards the null.^15,16^

### Selection of genetic variants

For each exposure, our instrumental variables included SNPs that are associated with the exposures at P-values ≤5×10^-8^ and are available in both the exposure and outcome GWASs. We selected independent SNPs by clumping, based on the European population in the 1000 genomes reference panel using an R^2^ threshold of 0.001 and excluded variants with minor allele frequency <5% in the exposure or outcome GWASs.

### Statistical analyses

We assessed the strength of our instrumental variables by calculating the average of SNP-specific F-statistics. We performed inverse-variance-weighted regression for the primary analysis, and simple and weighted median, and MR-Egger regression as sensitivity analyses using the R-package *MendelianRandomization*.^17^ Results of the primary analysis are presented as odds ratios (OR) with 95% confidence intervals (CIs). In addition, we performed MR-PRESSO to identify outlying SNPs that potentially introduce directional pleiotropy.^18^ Moreover, we used sex-combined instrumental variables for lipid traits and fasting glucose. Sex differences were quantified using Cochran’s Q-test.

Statistical analyses were conducted in R 4.1.2 (The R Foundation, Vienna, Austria). All statistical tests were two-sided and P-values ≤0.05 were deemed statistically significant.

## Results

### Overview of instrumental variables

An overview of the sex-specific instrumental variables is provided in Table 1. The majority of GWASs included data on individuals from European ancestry and included data from the UK Biobank. The number of SNPs included in each instrumental variable was similar for women and men and spread from 14 (instrumental variable for fasting glucose for men) to 250 (instrumental variables on HDL-C for both women and men). The average number of women and men included in the underlying GWAS was high, ranging from 57,842 to 684,030. In addition, the F-statistics ranged from 36.9 to 244.6.

### Relationship between genetically proxied cardiometabolic and lifestyle factors and risk of aSAH

**Figure 1:**
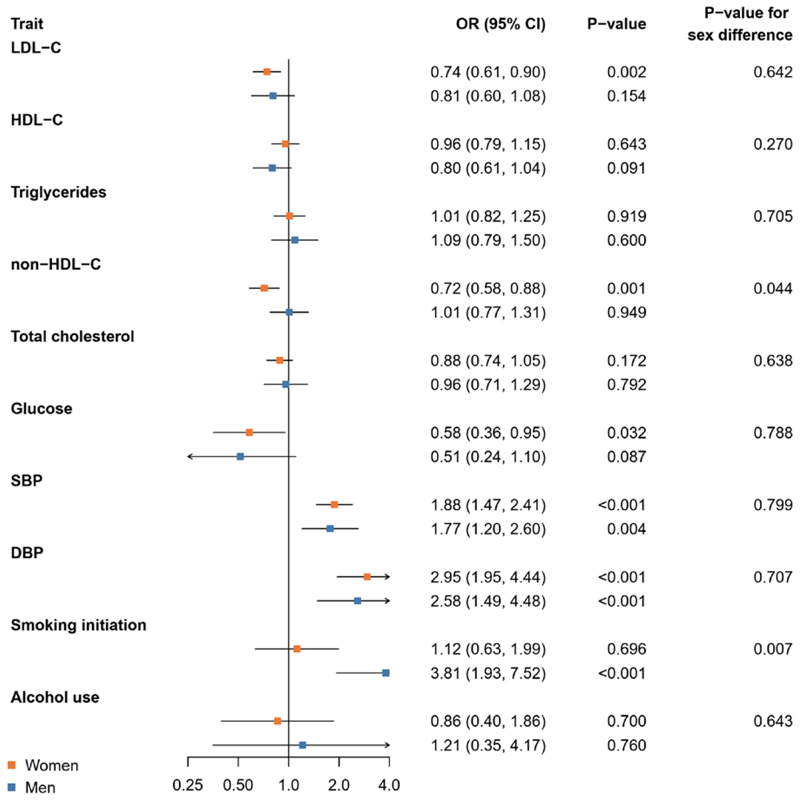
Mendelian randomisation analysis of genetically proxied cardiometabolic and lifestyle factors and risk of aneurysmal subarachnoid haemorrhage. Results are from inverse-variance weighted Mendelian randomisation. Abbreviations: CI, confidence interval; DBP, diastolic blood pressure; HDL-C, high-density lipoprotein cholesterol; LDL-C, low-density lipoprotein cholesterol; OR, odds ratio; SBP, systolic blood pressure.

Figure 1. shows the results of the Mendelian randomisation analysis for the risk of aSAH. Higher genetically proxied non-HDL-C was related to a statistically significant lower risk of aSAH in women (OR per standard deviation higher genetically proxied non-HDL-C 0.72 [95% CI 0.58, 0.88]) compared to men (1.01 [0.77, 1.31]) (P-value for sex difference 0.044). In addition, genetic liability to smoking initiation was associated with a statistically significantly higher risk of aSAH in men (OR per unit increase in log odds of genetic liability to smoking initiation 3.81 [1.93, 7.52]) compared to women (1.12 [0.63, 1.99]) (P-value for sex difference 0.007). Genetically proxied SBP and DBP were related to a higher risk of aSAH in both women and men without significant sex differences (P-values for sex differences ≥0.707).

### Relationship between genetically proxied cardiometabolic and lifestyle factors and risk of IA

**Figure 2:**
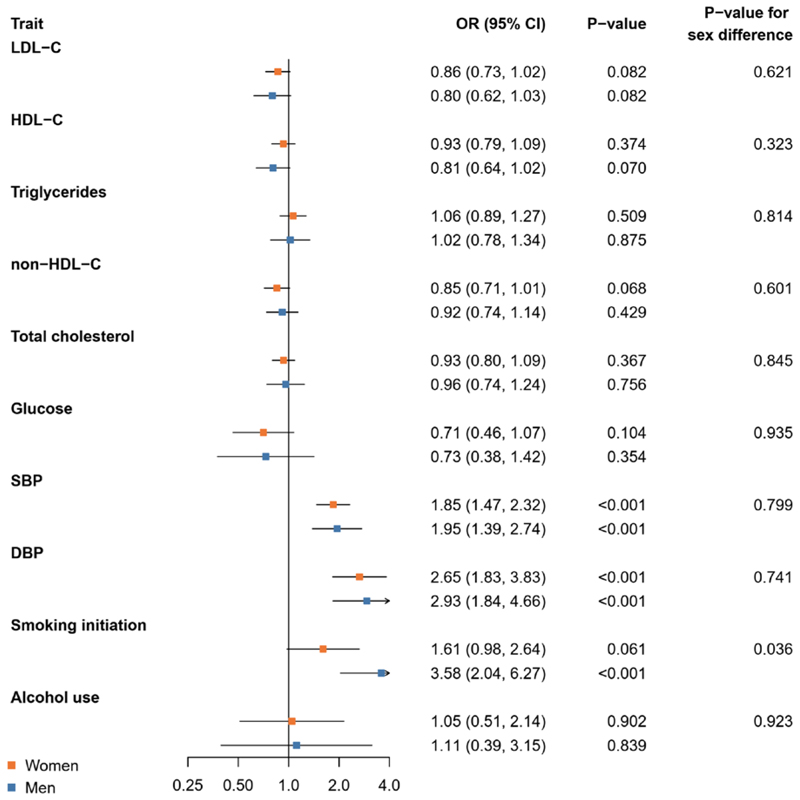
Mendelian randomisation analysis of genetically proxied cardiometabolic and lifestyle factors and risk of intracranial aneurysm. Results are from inverse-variance weighted Mendelian randomisation. Abbreviations: CI, confidence interval; DBP, diastolic blood pressure; HDL-C, high-density lipoprotein cholesterol; LDL-C, low-density lipoprotein cholesterol; OR, odds ratio; SBP, systolic blood pressure.

The Mendelian randomisation results for IA are depicted in Figure 2. We found no statistically significant sex difference in the risk of IA related to non-HDL-C with ORs of 0.85 (0.71, 1.01) in women and 0.92 (0.74, 1.14) in men per higher standard deviation in genetically proxied non-HDL-C (P-value for sex difference 0.323). The association with genetic liability to smoking initiation was statistically significantly different between the sexes with ORs of 3.58 (2.04, 6.27) and 1.61 (0.98, 2.64) in men and women, respectively, per unit increase in the log odds of genetic liability to smoking initiation (P-value for sex difference 0.036). Genetically proxied SBP and DBP were linked to a higher risk for IA in both women and men (P-values for sex differences ≥0.741).

### Sensitivity analyses

As shown in eFigure 1, eFigure 2, eFigure 3, and eFigure 4, results remained broadly consistent when applying different Mendelian randomisation methods. MR-Egger indicated potential directional pleiotropy for the relationship between genetically proxied DBP and risk of aSAH in men (P-value for MR-Egger intercept 0.027). As outlined in eTable 2, MR-PRESSO yielded significant outliers for some analyses. However, the distortion between the estimates before and after removing the significant outliers was only statistically significant when studying the association between genetically proxied triglycerides and IA in men, and both odds ratios were not statistically significantly different from one (OR 1.02 [0.78, 1.34] before removing outliers versus 0.99 [0.76, 1.29] after removing outliers). Results were directionally similar when using sex-combined instrumental variables for lipid and glucose traits (eFigure 5, eFigure 6).

## Discussion

In this Mendelian randomisation study, we found sex differences in the association between genetically proxied non-HDL-C and risk of aSAH, and between genetic liability to smoking initiation and risk of both aSAH and IA.

### Association between lipid traits and aSAH and IA

Our findings suggest that genetically proxied non-HDL-C is related to a lower aSAH risk in women as compared to men. The link between cholesterol and SAH has been studied previously, but studies reported a yet inconclusive relationship between cholesterol and aSAH. A prospective observational study of >30,000 individuals form Japan found no statistically significant association between non-HDL-C and aSAH in both sexes.^19^ Moreover, an observational meta-analysis of case-control studies reported a lower risk for SAH related to hypercholesterolaemia without significant sex differences.^5^ An observational analysis within the National FINRISK study reported hazard ratios for SAH of 1.15 (1.01, 1.31) in men and 1.04 (0.92, 1.18) in women per standard deviation higher LDL-C without a statistically significant interaction by sex.^20^ Previously conducted sex-combined Mendelian randomisation studies reported ORs of 0.89 (0.77, 1.02) for aSAH and of 0.90 (0.78, 1.02) per standard deviation higher genetically proxied LDL-C^6^ and an OR for IA of 0.83 (0.73, 0.94) per mmol higher genetically proxied LDL-C.^7^ Here, we provide additional context to the relationship between non-HDL-C and aSAH by showing that the causal risk-decreasing association appears to be stronger in women. The reasons for sex differences in the relationship between non-HDL-C and aSAH risk remain to be elucidated. Menopause and the drop in oestrogen levels is one of the factors that could potentially play a role in the development of aSAH. Incidence of aSAH is specifically high in women aged ≥55 years^21^ and sex-hormones have been related to aSAH risk.^22^ In addition, it has been hypothesised that oestrogens may affect blood vessel fragility,^23^ and a more fragile vessel wall can lead to aneurysm rupture.^24^ Moreover, we did not find a causal relationship between lipid traits and IA. Based on the available data, we were not able to study the relationship between genetically proxied lipid traits and risk of unruptured IA alone. However, genetically proxied non-HDL-C could be specifically related to the risk of aSAH, i.e., ruptured IAs, suggesting that non-HDL-C may only influence IA rupture and not development. In line with this hypothesis, a previous sex-combined Mendelian randomisation study reported higher genetically proxied LDL-C to be related to a lower risk of aSAH but not to the risk of unruptured IA.^7^

### Mechanisms of lipids in the development of aSAH

The underlying pathophysiological mechanisms of lipids in the development of aSAH are still unclear. It has been hypothesised that low cholesterol levels may lead to a more fragile cerebrovascular endothelium ultimately inducing aSAH.^24,25^ In our analysis, higher genetically proxied non-HDL-C was related to a lower risk for aSAH in women as compared to men. Non-HDL-C consists of several components including LDL-C, very low density lipoprotein, intermediate density lipoprotein, lipoprotein(a), and chylomicron remnants. Noteworthy, we found no statistically significant sex differences in the relationship between genetically proxied LDL-C and aSAH risk. A recent Mendelian randomisation study reported no relationship between genetically proxied intermediate and very low density lipoprotein and risk of SAH, however, they did not study sex differences.^26^ Understanding whether a specific component of non-HDL-C drives our findings would help to better understand pathophysiological mechanisms of lipids in the development of SAH. In addition, although not statistically significant, genetically proxied HDL-C tended towards a more protective relationship with risk for aSAH in men as compared to women, which could in part drive the finding for non-HDL-C.

In general, our findings may provide opportunities for potential new therapies of which women could benefit particularly, although it is essential to identify the right therapeutic target. It is important to note that, based on observational studies, use of lipid-lowering medication is more likely to be related to a lower risk of aSAH and IA. An observational analysis including 4,701 patients who were diagnosed with IA, showed use of lipid-lowering therapy to be inversely related to risk of IA rupture,^27^ but no sex-specific results were provided. In a case-control study from Japan, statin use was related to a lower risk of cerebral aneurysm rupture.^28^ Furthermore, in a case-control study within the PHARMO database, a Dutch population based system with dispensing data, current use compared to no use of statins was related to a lower risk of SAH, while recent withdrawal of statins compared to current use was related to a higher risk of SAH.^29^ Consequently, in order to develop new therapies, it is crucial to better understand the role of lipids and lipid-lowering medication in the risk of developing aSAH and IA.

### Association between smoking and aSAH and IA

Our analyses yielded statistically significant sex differences in the relationship between genetic liability to smoking initiation and risk of aSAH and IA with a higher risk in men as compared to women. A previously conducted sex-combined Mendelian randomisation study also reported a causal relationship between smoking traits and higher risk of aSAH and IA.^6^ We now show here that the higher risk for aSAH and IA is more pronounced in men. Our Mendelian randomisation results are not in line with observational findings. A meta-analysis of 14 longitudinal and 23 case-control studies reported smoking to be related to a higher risk of SAH in both women and men.^5^ For instance, for ever smoking, the relative risk for SAH was 2.6 (2.0, 3.5) in women and 3.4 (2.4, 4.7) for men in case-cohort studies.^5^ In longitudinal studies, the relative risk for SAH related to ever smoking was only statistically significant in women with a relative risk of 2.7 (1.8, 4.1), while the relative risk for SAH in men was 1.4 (0.9, 2.1).^5^ Another observational meta-analysis formally tested for heterogeneity between the sexes and found no significant sex differences in the association between current and former smoking and risk of aSAH.^8^ The discrepancy between observational and Mendelian randomisation studies could have various reasons. On the one hand, observational results could be affected by confounding, and the Mendelian randomisation provides –under certain assumptions– unconfounded results. On the other hand, it needs to be noted that our instrumental variables may not capture sex-specific smoking initiation. Indeed, we used sex-combined instrumental variables for smoking initiation, as –to the best of our knowledge– no sex-specific GWAS on smoking has been published so far. Thus, it is unclear how sex-specific genetic instruments would have affected our findings. In addition, given that men smoke on average more frequently than women, our genetic instruments for smoking initiation could be biased towards being more closely related to phenotypic smoking in men. Consequently, our findings on genetically proxied smoking initiation need to be interpreted with caution and future sex-specific GWASs are needed to study sex-specific instrumental variables for smoking initiation.

### Association between other cardiometabolic and lifestyle factors and aSAH and IA

In our Mendelian randomisation study, we found significant associations between genetically proxied SBP and DBP and higher risk for both aSAH and IA without significant sex differences. Results from a previous sex-combined Mendelian randomisation study are highly comparable.^6^ For instance, higher genetically proxied SBP was related to an OR for IA of 1.87 (1.61, 2.17) in sex-combined analysis,^6^ while we found ORs for IA of 1.85 (1.47, 2.32) in women and 1.95 (1.39, 2.74) in men. The same sex-combined study also reported a significant relationship between higher genetically proxied fasting glucose and risk of aSAH and IA.^6^ In our study, the relationship between genetically proxied fasting glucose and aSAH and IA tended towards the same direction. We found no relationship between genetic liability to alcohol use and the risk of aSAH or IA in both sexes. Contrarily, a sex-combined Mendelian randomisation study in the UK Biobank found a significantly higher risk for SAH per standard deviation increase in log-transformed drinks of alcohol per week.^30^ An important difference is that this study included data from the UK Biobank when studying genetic associations with both the exposure and the outcome. Contrarily, we have excluded data from the UK Biobank from the dataset including genetic associations with aSAH and IA to avoid sample overlap in the current analysis, which minimises bias towards to confounded result. Moreover, in an observational meta-analysis, each additional 10 g of alcohol per day were related to a higher risk for SAH of 12.1%.^31^

### Strengths and limitations

Our study has several strengths. We included a data on genetic associations with a broad range of cardiometabolic and lifestyle factors from large-scale GWASs. Moreover, we used sex-specific genetic associations with lipid and glucose traits. In addition, we performed a variety of sensitivity analyses to study the robustness of our findings. Our study also has limitations. Mendelian randomisation studies rely on three assumptions. It is assumed that the genetic instrument (1) is associated to the exposure, (2) is not associated to confounding factors, and (3) can influence the outcome only via the exposure. To assess the strength of our genetic instrument, we calculated F-statistics for all instrumental variables, which were sufficiently high (>10 as suggested by Staiger and Stock^32^). In addition, we conducted sensitivity analyses to investigate directional pleiotropy. We performed MR-Egger and found a statistically significant intercept in the association between genetically proxied DBP and risk of IA in men. However, MR-PRESSO did not yield any significant outlying SNPs for this relationship. Although we thoroughly checked the assumptions of Mendelian randomisation, we still cannot rule out violation of any of the criteria. Another limitation is that we used sex-combined genetic associations with blood pressure, smoking and alcohol traits, making it unclear whether the corresponding genetic instruments are adequate for both sexes. Furthermore, genetic associations with SBP and DBP included individuals from multiple ancestries, while the outcome GWAS was based on data from individuals of European ancestry.

## Conclusions

This Mendelian randomisation study revealed potential sex differences in relationships of genetically proxied cardiometabolic and lifestyle factors with aSAH and IA. Higher genetically proxied non-HDL-C was related to a statistically significantly lower aSAH risk in women compared to men. Moreover, genetic liability to smoking initiation was associated with a statistically significantly higher aSAH and IA risk in men compared to women. These findings may help understand sex differences in the development of aSAH and IA.

## Supplementary Material

Supplementary Materials

## Figures and Tables

**Table 1:** Sex-specific instrumental variables used for Mendelian randomisation analysis.

					Women			Men	
Cardiometabolic/lifes tyle factor (unit)	Data source	UKB incl.	Ancestry	No. of SNPs	N^a^	F^b^	No. of SNPs	N^a^	F^b^
LDL-C (SD)	GLGC^10^	Yes	EUR	203	555,370	223.1	197	671,163	197.1
HDL-C (SD)	GLGC^10^	Yes	EUR	250	563,169	184.1	250	677,684	202.8
Triglycerides (SD)	GLGC^10^	Yes	EUR	218	566,795	149.3	213	684,030	174.5
Non-HDL-C (SD)	GLGC^10^	Yes	EUR	197	482,421	166.2	151	399,638	190.4
Total cholesterol (SD)	GLGC^10^	Yes	EUR	228	604,672	244.6	215	710,840	192.0
Glucose (1 mmol/L)	MAGIC^11^	No	EUR	19	63,548	96.6	14	57,842	104.8
SBP (10 mmHg)	ICBP^12^	No	Mixed	37	192,857	56.7	37	192,857	56.7
DBP (10 mmHg)	ICBP^12^	No	Mixed	39	191,828	51.0	39	191,828	51.0
Smoking initiation (ever vs never)	GSCAN^13^	Yes	EUR	198	797,006	37.0	199	797,048	36.9
Alcohol use (drinks per week)	GSCAN^13^	Yes	EUR	71	662,724	42.3	71	662,724	42.3

a The sample size N is based the average across sample sizes for all SNPs included in the instrumental variables.

b The F-statistics were calculated for each SNP and averaged over all SNPs. Number of SNPs and F-statistics were the same for aneurysmal subarachnoid haemorrhage and intracranial aneurysm. Abbreviations: DBP, diastolic blood pressure; EUR, European; GLGC, Global Lipids Genetics Consortium; GSCAN, Genome-wide association study and Sequencing Consortium of Alcohol and Nicotine use; HDL-C, high-density lipoprotein cholesterol; ICBP, International Consortium for Blood Pressure; LDL-C, low-density lipoprotein cholesterol; MAGIC, Meta-Analyses of Glucose and Insulin-related traits Consortium; SBP, systolic blood pressure; SD, standard deviation; SNP, single nucleotide polymorphism; UKB, UK Biobank.

## Data Availability

Data on genetic associations with LDL-C, HDL-C, triglycerides, non-HDL-C, total cholesterol, fasting glucose, SBP, DBP, smoking initiation, and alcohol use are publicly available. Sex-specific genetic associations with aSAH and IA can be made available from YMR upon reasonable request.
